# Association of nonalcoholic fatty liver disease and liver fibrosis detected by transient elastography with serum retinol in American adults

**DOI:** 10.3389/fnut.2023.1094161

**Published:** 2023-03-15

**Authors:** Xiaoxian Niu, Jian Liu, Kai Liu

**Affiliations:** ^1^Department of Ultrasound, The First Affiliated Hospital of Henan Polytechnic University (Jiaozuo Second People's Hospital), Jiaozuo, China; ^2^Department of Oncology, The First Affiliated Hospital of Henan Polytechnic University (Jiaozuo Second People's Hospital), Jiaozuo, China

**Keywords:** NAFLD, liver fibrosis, serum retinol, NHANES, transient elastography

## Abstract

**Background and objective:**

Retinol is a precursor of vitamin A, which is metabolized and maintained in the liver and is involved in the pathogenesis of the nonalcoholic fatty liver disease (NAFLD) and liver fibrosis. The relationship between NAFLD and liver fibrosis with serum retinol levels remains insufficient and inconclusive. Our study aimed to investigate the correlation between NAFLD, fibrosis, and serum retinol levels in American adults.

**Methods:**

A cross-sectional analysis was conducted using information from the 2017–2018 cycle of the National Health and Nutrition Examination Survey (NHANES). The exposure factors were NAFLD and liver fibrosis status detected using transient elastography (TE), and the outcome was serum retinol levels. Weighted multivariate regressions were established to assess the correlation between NAFLD and liver fibrosis and serum retinol levels. Subgroup analyses were also performed.

**Results:**

This study included 3,537 participants. Compared to the group without NAFLD, NAFLD was positively correlated with serum retinol levels (β = 1.28, 95% CI: 0.19, 2.37). In the subgroup analysis, a positive correlation between NAFLD and serum retinol levels was found in people aged < 60 years, Mexican Americans, and those with a body mass index (BMI) < 25. On the contrary, compared to the group without liver fibrosis, there was a significant negative association between liver fibrosis and serum retinol (β = −3.46, 95% CI: −5.16, −1.75), especially in people aged < 60 years, non-Hispanic white/black individuals, and people with a BMI ≥ 25.

**Conclusion:**

Our study suggests that NAFLD status may be positively associated with serum retinol levels in adult patients, and liver fibrosis may be negatively associated with serum retinol levels. Further studies are required to examine the associations found in our study.

## 1. Introduction

Nonalcoholic fatty liver disease (NAFLD) is a common chronic liver disease that affects one-third of American adults, resulting in a substantial disease burden (1). NAFLD is a fatty liver disease in which excessive fat is stored in liver cells (2, 3). It is an umbrella term that comprises a continuum of liver conditions that vary in the severity of the injury and resulting fibrosis (4). NAFLD has a range of pathological changes (5) including isolated steatosis and progressive nonalcoholic steatohepatitis. Approximately 40% of patients with nonalcoholic steatohepatitis eventually develop liver fibrosis, cirrhosis, and hepatocellular carcinoma (6). Because there is no effective treatment for NAFLD and fibrosis, it is necessary to identify the hazardous factors that contribute to disease development.

Dietary vitamin A is absorbed in the small intestine and transported to the liver in the form of retinyl esters, which are then stored in hepatic stellate cells (HSCs) (7). Retinol binds to retinol-binding protein 4 (RBP4) produced by hepatocytes and is secreted into the circulation, where it plays a physiological role. Vitamin A is a key regulator of glucose and lipid metabolism in the liver and adipose tissue (8). It has been suggested that disturbances in vitamin A homeostasis in the liver may contribute to the development of NAFLD (9). Specifically, changes in diet or hormonal signaling can activate HSCs, leading to the loss of the ability of HSCs to store vitamin A. Excess vitamin A metabolism increases lipogenesis, allowing fat to accumulate in hepatocytes, eventually inducing NAFLD. After a long-term chronic liver injury, HSCs transdifferentiate into myofibroblasts and produce an excessive extracellular matrix, leading to liver fibrosis. HSCs lose their stored retinyl esters during this process, eventually leading to a vitamin A deficiency.

Retinol is a precursor of vitamin A, and the serum retinol level is a sensitive marker for the evaluation of vitamin A status (10). However, reports on the epidemiological relationship between NAFLD and liver fibrosis and serum retinol levels are scarce, and the results of studies on serum vitamin A levels in patients with NAFLD or fibrosis are controversial (11–13).

Liver ultrasound transient elastography (TE) is a noninvasive method for estimating liver steatosis and fibrosis (14) and has been used in the general population (15). The National Health and Nutritional Examination Survey (NHANES) first used TE examinations during the 2017–2018 cycle. Here, we aimed to investigate the clinical relevance of serum retinol levels in the setting of NAFLD and liver fibrosis detected by TE in adults and to provide a new perspective on its pathogenesis.

## 2. Materials and methods

The NHANES is a cross-sectional survey conducted in the United States based on a nationally representative sample. Our study used data from the 2017–2018 NHANES cycle, in which the TE examination was first used.

### 2.1. Variables

The exposure factors were NAFLD status detected by the control attenuation parameter (CAP) and liver fibrosis status detected by liver stiffness measurement (LSM). CAP and LSM values were implemented on FibroScan®, an instrument that can use the vibration-controlled transient elastography (VCTE) technique, obtained in mobile examination centers (MEC). Qualified VCTE tests required the following three aspects: fasting for at least 3 h, 10 LSM measurements were obtained at least, and an interquartile range (IQR)/median of <30%. Liver steatosis was defined as a median CAP value ≥ 274 dB/m (16) based on a recent study. Significant fibrosis (≥F2) was defined as a median LSM value ≥ 8.0 kPa (17, 18). The outcome was the serum retinol level, detected using a modification of a high-performance liquid chromatography-photodiode array detection method, as recommended by the NHANES website.^1^ Specifically, the serum was mixed with an ethanol solution containing an internal standard, retinyl butyrate. After extracting the micronutrients, an aliquot of the filtrate was injected into a C18 reversed-phase column. Quantitative analysis was performed using spectrophotometry. Retinol and retinyl esters were compared with retinyl butyrate at 325 nm.

The covariates included age, race, sex, waist circumference, body mass index (BMI), triglyceride, total cholesterol, alanine aminotransferase (ALT), aspartate transaminase (AST), γ-glutamyl transpeptidase (GGT), total bilirubin, serum albumin, serum creatinine, blood urea nitrogen, recreational physical activity, smoking status, and diabetes status. All data are available on the NHANES’s website. The participants were classified as never, former, or current smokers. Participants were considered never smokers if they had smoked <100 cigarettes and former or current smokers based on whether they smoked currently or not, or if they had ever smoked ≥ 100 cigarettes. Diabetes was defined based on the guidelines for the Classification and Diagnosis of Diabetes. HBsAg and anti-HCV antibodies were used to detect the hepatitis B virus (HBV) and hepatitis C virus (HCV), respectively. Alcohol intake data were obtained from the questionnaire according to alcohol consumption in the previous year. Significant alcohol intake was defined as >21 or > 14 standard drinks weekly for men or women (19).

### 2.2. Study population

**Figure 1:**
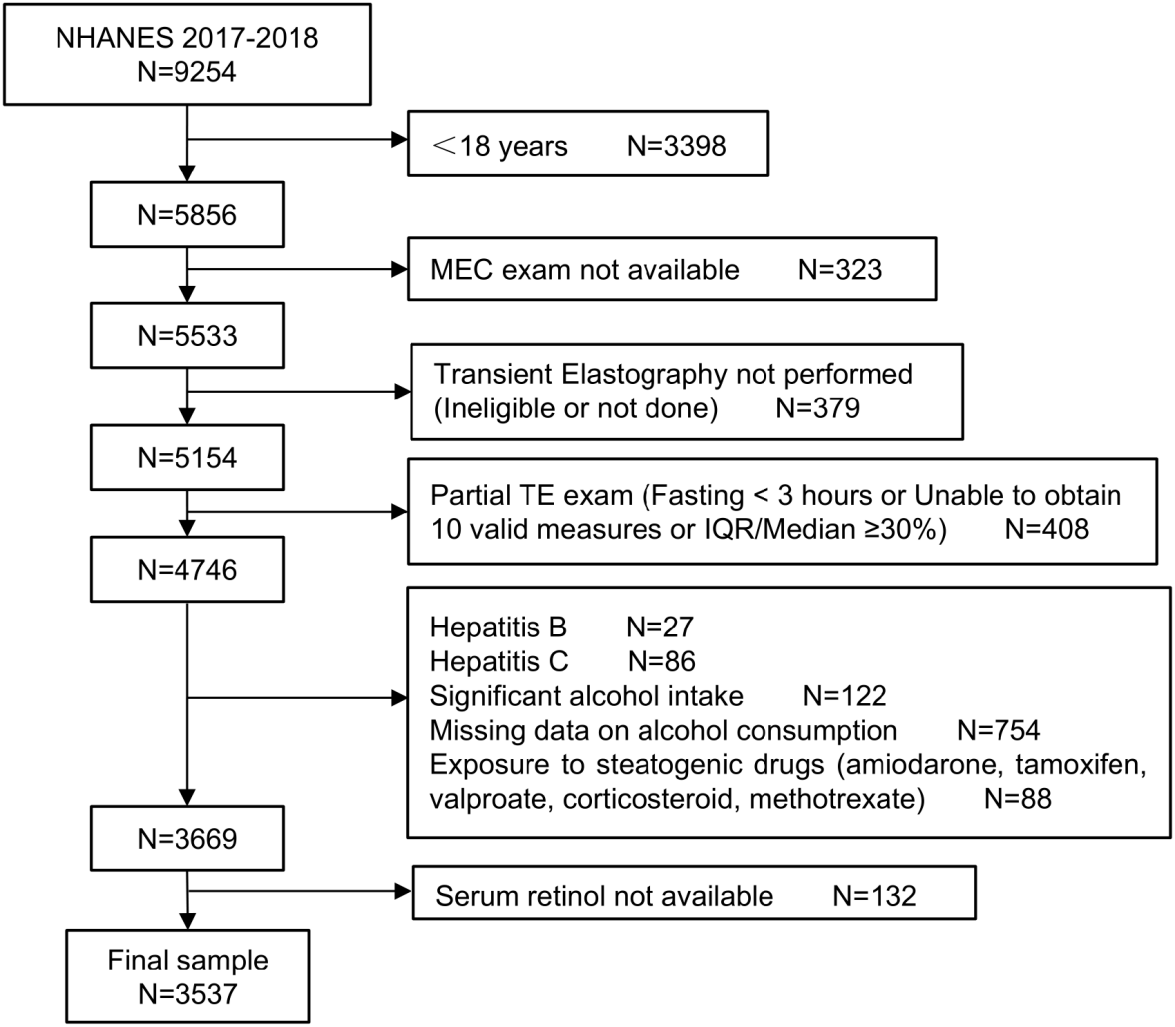
Flowchart of the study participants.

In total, 9,254 participants were included in the 2017–2018 NHANES cycle. We excluded the following participants: individuals aged <18 years, individuals whose MEC exam was not available, individuals for whom a transient elastography was not performed (ineligible or not done), individuals for whom only a partial TE exam was performed (fasting < 3 h or incapable of obtaining at least 10 effective measures or IQR/Median ≥ 30%), individuals with Hepatitis B and C, individuals with significant alcohol intake or missing alcohol use data, individuals with exposure to steatogenic drugs (amiodarone, tamoxifen, corticosteroid, valproate, and methotrexate), and individuals whose serum retinol levels were not available. Finally, 3,537 samples were analyzed (Figure 1).

### 2.3. Statistical analysis

Appropriate sample weights were used for analysis. Data were presented as weighted proportions and weighted mean ± standard error (SE) for different types of covariables. We used weighted chi-square tests and linear regression models to explore differences between patients with NAFLD and fibrosis and those without NAFLD and fibrosis. Multifactor linear regression models were used to assess the correlation between NAFLD and fibrosis status with serum retinol levels.

Three models were created in the study, as recommended by the guidelines for reporting observational studies (20): Model 1, no adjusted covariables; Model 2, adjustments made for sex, race, and age; Model 3, in addition to the variables in Model 2, waist circumference, BMI, total cholesterol, triglyceride, ALT, AST, GGT, total bilirubin, serum albumin, serum creatinine, blood urea nitrogen, recreational physical activity, smoking status, and diabetes status were added. Subgroup analyses were performed based on age, sex, race, and BMI.

EmpowerStats^2^ and R^3^ software packages were used for the analyses. *p*-values of <0.05 were defined as statistically significant.

## 3. Results

### 3.1. Population characteristics

A total of 3,537 samples were included in the study. The weighted characteristics of the samples according to NAFLD and fibrosis status are shown in Table 1. There were significant differences in sample characteristics among patients with different NAFLD statuses. Compared with the group without NAFLD, the patients with NAFLD were more likely to be older, men, non-Hispanic white, and former smokers. They also had higher BMIs, waist circumference, total cholesterol, triglyceride, ALT, AST, GGT, blood urea nitrogen, and LSM values, and lower total bilirubin and serum albumin levels. Moreover, they were less likely to undergo recreational physical activity and had a higher prevalence of diabetes (*p* < 0.05). Weighted characteristics based on liver fibrosis showed similar results. In brief, there were significant differences in most sample characteristics (Table 1), except for race, total cholesterol, total bilirubin, and blood urea nitrogen.

**Table 1:** Weighted characteristics of the study sample based on NAFLD and liver fibrosis status.

Characteristic	Total	NAFLD		*p*-value	Liver fibrosis		*p*-value
		No	Yes		No	Yes	
CAP < 274 dB/m	CAP ≥ 274 dB/m	LSM < 8.0 kPa	LSM ≥ 8.0 kPa
N	3,669	1983	1,554		3,186	351	
Age (years)	46.92 ± 17.42	44.10 ± 17.61	50.90 ± 16.33	<0.0001	46.57 ± 17.40	50.86 ± 17.17	<0.0001
Gender (%)				<0.0001			<0.0001
Male	49.45	44.99	55.76		48.43	60.86	
Female	50.55	55.01	44.24		51.57	39.14	
Race (%)				<0.0001			0.8006
Mexican American	9.47	6.83	13.20		9.34	10.93	
Other Hispanic	7.07	7.39	6.61		6.98	8.05	
Non-Hispanic white	63.94	65.18	62.18		64.05	62.68	
Non-Hispanic Black	10.41	11.87	8.34		10.51	9.24	
Other Race	9.11	8.73	9.67		9.12	9.10	
BMI	29.61 ± 7.05	26.70 ± 5.62	33.70 ± 6.83	<0.0001	28.95 ± 6.39	36.86 ± 9.55	<0.0001
Waist circumference (cm)	100.19 ± 17.02	92.47 ± 13.94	111.08 ± 14.88	<0.0001	98.59 ± 15.77	117.94 ± 19.97	<0.0001
Total cholesterol (mg/dL)	188.27 ± 40.62	186.40 ± 39.66	190.91 ± 41.79	0.0011	188.69 ± 40.24	183.61 ± 44.38	0.0407
Triglyceride (mg/dL)	112.78 ± 71.82	101.78 ± 42.24	128.32 ± 97.52	<0.0001	111.33 ± 66.43	128.93 ± 114.65	<0.0001
ALT (IU/L)	27.53 ± 18.52	19.23 ± 13.56	27.52 ± 18.57	<0.0001	21.81 ± 15.32	32.20 ± 22.99	<0.0001
AST (IU/L)	21.51 ± 10.93	20.52 ± 9.88	22.92 ± 12.13	<0.0001	21.02 ± 9.68	27.06 ± 19.31	<0.0001
GGT (IU/L)	28.16 ± 36.32	23.09 ± 33.35	35.33 ± 39.03	<0.0001	26.12 ± 27.83	50.90 ± 82.51	<0.0001
Total bilirubin (mg/dL)	0.47 ± 0.28	0.49 ± 0.29	0.45 ± 0.26	<0.0001	0.47 ± 0.28	0.47 ± 0.25	0.9629
Serum albumin (g/dL)	4.11 ± 0.32	4.13 ± 0.31	4.07 ± 0.32	<0.0001	4.11 ± 0.31	4.04 ± 0.36	0.0002
Serum creatinine (mg/dL)	0.88 ± 0.31	0.87 ± 0.24	0.89 ± 0.39	0.0649	0.88 ± 0.30	0.93 ± 0.43	0.0058
Blood urea nitrogen (mg/dL)	14.95 ± 5.06	14.55 ± 4.83	15.51 ± 5.33	<0.0001	14.90 ± 5.03	15.50 ± 5.40	0.0526
Recreational physical activity (%)				<0.0001			<0.0001
Yes	56.89	63.35	47.78		58.24	41.94	
No	43.11	36.65	52.22		41.76	58.06	
Smoking status (%)				0.0002			0.0125
Never	58.01	59.88	55.37		58.53	52.25	
Former	25.45	22.92	29.02		24.80	32.67	
Current	16.54	17.20	15.62		16.67	15.08	
Diabetes (%)				<0.0001			<0.0001
Yes	13.91	5.99	25.08		11.45	41.23	
No	86.09	94.01	74.92		88.55	58.77	
Serum retinol (μg/dL)	54.52 ± 15.66	53.34 ± 15.18	56.19 ± 16.16	<0.0001	54.64 ± 15.64	53.18 ± 15.79	0.1269
CAP (dB/m)	261.97 ± 62.41	219.17 ± 36.08	322.41 ± 36.30	<0.0001	256.78 ± 59.85	319.59 ± 61.31	<0.0001
LSM (kPa)	5.62 ± 4.87	4.82 ± 2.99	6.75 ± 6.50	<0.0001	4.79 ± 1.24	14.81 ± 13.33	<0.0001

Mean ± SD and % for continuous and categorical variables. The *P*-value was calculated by weight linear regression model and weighted chi-square test, respectively.

### 3.2. Relationship between NAFLD and serum retinol

We constructed three weighted linear regression models as shown in Table 2. Compared to the group without NAFLD, there was a significant positive correlation between the NAFLD group and serum retinol in Model 1 (β = 2.85, 95% CI: 1.81, 3.89). After adjusting for covariates, a significant positive correlation was still present in Model 2 (β = 1.04, 95% CI: 0.02 2.06) and Model 3 (β = 1.28, 95% CI: 0.19, 2.37). In further subgroup analyses (Table 3) stratified by age, sex, race, and body mass index (BMI), a positive correlation remained in people aged < 60 years (β = 1.40, 95% CI: 0.07, 2.72), Mexican Americans (β = 2.47, 95% CI: 0.18, 4.77), and those with a BMI < 25 (β = 6.48, 95% CI: 3.10, 9.86).

**Table 2:** Relationship between NAFLD or liver fibrosis status and serum retinol (μg/dL) in adults.

	Model 1	Model 2	Model 3
	β (95%CI) *p*-value	β (95%CI) *p*-value	β (95%CI) *p*-value
**NAFLD status**			
No	Reference	Reference	Reference
Yes	2.85 (1.81, 3.89) <0.0001	1.04 (0.02, 2.06) 0.0453	1.28 (0.19, 2.37) 0.0218
**Liver fibrosis status**			
No	Reference	Reference	Reference
Yes	−1.46 (−3.33, 0.41) 0.1269	−3.06 (−4.83, −1.29) 0.0007	−3.46 (−5.16, −1.75) <0.0001

Model 1, no adjusted covariables; Model 2, adjustments made for gender, race, and age; Model 3, in addition to the variables in Model 2, BMI, waist circumference, total cholesterol, triglyceride, ALT, AST, GGT, total bilirubin, serum albumin, serum creatinine, blood urea nitrogen, recreational physical activity, smoking status, and diabetes were added. In different stratified policies, stratified variables were not adjusted themselves.

**Table 3:** Relationship between NAFLD status and serum retinol (μg/dL) in adults, stratified by age, gender, race, and BMI.

NAFLD status	Model 1	Model 2	Model 3
	β (95%CI) *p*-value	β (95%CI) *p*-value	β (95%CI) *p*-value
**<60 years**			
No	Reference	Reference	Reference
Yes	2.16 (0.91, 3.40) 0.0007	1.53 (0.34, 2.72) 0.0121	1.40 (0.07, 2.72) 0.0397
**≥60 years**			
No	Reference	Reference	Reference
Yes	2.72 (0.83, 4.61) 0.0049	2.75 (0.85, 4.65) 0.0046	1.60 (−0.24, 3.44) 0.0887
**Male**			
No	Reference	Reference	Reference
Yes	2.26 (0.84, 3.67) 0.0018	1.35 (−0.08, 2.77) 0.0638	1.33 (−0.24, 2.90) 0.0966
**Female**			
No	Reference	Reference	Reference
Yes	2.21 (0.70, 3.72) 0.0041	0.63 (−0.83, 2.10) 0.3970	1.44 (−0.08, 2.97) 0.0636
**Mexican American**			
No	Reference	Reference	Reference
Yes	3.36 (0.98, 5.74) 0.0058	0.68 (−1.56, 2.91) 0.5537	2.47 (0.18, 4.77) 0.0353
**Other Hispanic**			
No	Reference	Reference	Reference
Yes	0.74 (−2.14, 3.61) 0.6161	−0.94 (−3.72, 1.84) 0.5079	0.59 (−2.29, 3.46) 0.6899
**Non-Hispanic white**			
No	Reference	Reference	Reference
Yes	2.66 (0.90, 4.43) 0.0031	0.67 (−1.09, 2.43) 0.4539	1.41 (−0.50, 3.32) 0.1491
**Non-Hispanic Black**			
No	Reference	Reference	Reference
Yes	5.21 (2.89, 7.52) <0.0001	3.05 (0.80, 5.30) 0.0081	0.76 (−1.59, 3.10) 0.5258
**Other Race**			
No	Reference	Reference	Reference
Yes	4.78 (2.43, 7.13) <0.0001	3.05 (0.75, 5.36) 0.0097	0.18 (−2.16, 2.52) 0.8792
**BMI < 25**			
No	Reference	Reference	Reference
Yes	11.33 (7.88, 14.79) <0.0001	8.46 (5.01, 11.91) <0.0001	6.48 (3.10, 9.86) 0.0002
**BMI 25–29.9**			
No	Reference	Reference	Reference
Yes	1.37 (−0.49, 3.24) 0.1493	−0.09 (−1.92, 1.74) 0.9244	−0.36 (−2.08, 1.36) 0.6826
**BMI ≥ 30**			
No	Reference	Reference	Reference
Yes	3.41 (1.69, 5.14) 0.0001	1.79 (0.12, 3.46) 0.0354	1.53 (−0.02, 3.07) 0.0528

Model 1, no adjusted covariables; Model 2, adjustments made for gender, race, and age; Model 3, in addition to the variables in Model 2, BMI, waist circumference, total cholesterol, triglyceride, ALT, AST, GGT, total bilirubin, serum albumin, serum creatinine, blood urea nitrogen, recreational physical activity, smoking status, and diabetes were added. In different stratified policies, stratified variables were not adjusted themselves.

### 3.3. Relationship between liver fibrosis and serum retinol

In terms of the relationship between liver fibrosis status and serum retinol, compared with the group without liver fibrosis (Table 3), no significant correlation was found in Model 1 (β = −1.46, 95% CI: −3.33, 0.41) in the liver fibrosis group. Interestingly, there was a significant negative correlation after adjusting for the covariates in Model 2 (β = −3.06, 95% CI: −4.83, −1.29) and Model 3 (β = −3.46, 95% CI: −5.16, −1.75). In subgroup analyses (Table 4), the positive correlation remained in people aged < 60 years (β = −3.74, 95% CI: −5.89, −1.58), both men (β = −3.57, 95% CI: −5.79, −1.35) and women (β = −3.45, 95% CI: −6.13, −0.76), non-Hispanic white individuals (β = −3.47, 95% CI: −6.42, −0.52), non-Hispanic black individuals (β = −6.22, 95% CI: −9.87, 2.58), and those with a BMI of 25–29.9 (β = −4.77, 95% CI: −8.96, −0.59) and a BMI ≥ 30 (β = −2.87, 95% CI: −4.90, −0.85).

**Table 4:** Relationship between liver fibrosis status and serum retinol (μg/dL) in adults, stratified by age, gender, race, and BMI.

Liver fibrosis status	Model 1	Model 2	Model 3
	β (95%CI) *p*-value	β (95%CI) *p*-value	β (95%CI) *p*-value
**<60 years**			
No	Reference	Reference	Reference
Yes	−2.96 (−5.27, −0.64) 0.0123	−4.18 (−6.36, −1.99) 0.0002	−3.74 (−5.89, −1.58) 0.0007
**≥60 years**			
No	Reference	Reference	Reference
Yes	−0.01 (−3.09, 3.07) 0.9957	0.26 (−2.82, 3.35) 0.8670	−2.75 (−5.50, 0.00) 0.0504
**Male**			
No	Reference	Reference	Reference
Yes	−2.82 (−5.16, −0.48) 0.0182	−3.57 (−5.85, −1.29) 0.0022	−3.57 (−5.79, −1.35) 0.0016
**Female**			
No	Reference	Reference	Reference
Yes	−1.30 (−4.26, 1.67) 0.3910	−2.24 (−5.04, 0.56) 0.1171	−3.45 (−6.13, −0.76) 0.0120
**Mexican American**			
No	Reference	Reference	Reference
Yes	−0.86 (−4.88, 3.17) 0.6767	−3.83 (−7.55, −0.11) 0.0440	−0.85 (−4.40, 2.70) 0.6385
**Other Hispanic**			
No	Reference	Reference	Reference
Yes	−0.86 (−4.88, 3.17) 0.6767	−3.83 (−7.55, −0.11) 0.0440	−0.85 (−4.40, 2.70) 0.6385
**Non-Hispanic white**			
No	Reference	Reference	Reference
Yes	−2.02 (−5.19, 1.16) 0.2138	−3.18 (−6.26, −0.11) 0.0428	−3.47 (−6.42, −0.52) 0.0213
**Non-Hispanic Black**			
No	Reference	Reference	Reference
Yes	0.97 (−3.27, 5.21) 0.6541	−2.14 (−6.18, 1.90) 0.2999	−6.22 (−9.87, −2.58) 0.0009
**Other Race**			
No	Reference	Reference	Reference
Yes	3.17 (−1.12, 7.46) 0.1478	1.00 (−3.11, 5.11) 0.6327	−2.38 (−6.40, 1.64) 0.2470
**BMI < 25**			
No	Reference	Reference	Reference
Yes	1.41 (−4.44, 7.26) 0.6362	−0.90 (−6.50, 4.69) 0.7518	−2.37 (−7.93, 3.19) 0.4030
**BMI 25–29.9**			
No	Reference	Reference	Reference
Yes	−1.36 (−6.15, 3.43) 0.5767	−4.09 (−8.64, 0.46) 0.0786	−4.77 (−8.96, −0.59) 0.0257
**BMI ≥ 30**			
No	Reference	Reference	Reference
Yes	−1.34 (−3.60, 0.92) 0.2441	−2.41 (−4.55, −0.28) 0.0271	−2.87 (−4.90, −0.85) 0.005

Model 1, no adjusted covariables; Model 2, adjustments made for gender, race, and age; Model 3, in addition to the variables in Model 2, BMI, waist circumference, total cholesterol, triglyceride, ALT, AST, GGT, total bilirubin, serum albumin, serum creatinine, blood urea nitrogen, recreational physical activity, smoking status, and diabetes were added. In different stratified policies, stratified variables were not adjusted themselves.

## 4. Discussion

In our study, the 2017–2018 NHANES cycle was used to examine the correlation between NAFLD and fibrosis status detected by TE with serum retinol in adults. NAFLD was positively associated with serum retinol levels, especially in those aged <60 years, Mexican Americans, and those with a BMI < 25. In contrast, liver fibrosis status was negatively associated with serum retinol levels, especially in people aged <60 years, non-Hispanic white/black individuals, and those with a BMI ≥ 25.

Vitamin A is necessary for glucose and lipid metabolism (21, 22) in the liver and adipose tissue (13), even in patients with NAFLD (23, 24). Retinol has an important antioxidant effect on NAFLD (25, 26). However, it remains unclear whether vitamin A levels promote or inhibit NAFLD development. Serum vitamin A levels in patients with NAFLD are controversial. A cross-sectional study reported that serum vitamin A levels were negatively correlated with NAFLD severity (11). Another study reported that serum retinol levels are inadequate in NAFLD (27). Nevertheless, another study indicated that serum retinol levels were higher in NAFLD donors than in non-NAFLD donors (12). A recent study from South Korea (13) showed that serum retinol levels were positively associated with the prevalence of NAFLD. According to the results of our research, patients with NAFLD had higher serum retinol levels than those without NAFLD, especially in those aged < 60 years, Mexican Americans, and with a BMI < 25.

Liver fibrosis has a significant effect on vitamin A metabolism and storage. HSCs contain approximately 80% retinol. HSCs are activated to produce large amounts of collagen and fibroblasts under oxidative stress, eventually leading to fibrosis (7). Vitamin A reserves are also gradually lost during this conversion process (25). In most NAFLD prediction models, low serum retinol levels were significantly associated with advanced fibrosis. According to a recent study (13), serum retinol deficiency was significantly associated with advanced fibrosis. Another study showed that reduced serum retinol levels were observed in patients with advanced liver fibrosis, and liver fibrosis was an independent risk factor correlated with decreased serum retinol levels (27). Our study showed that compared with the group without liver fibrosis, patients with liver fibrosis had lower serum retinol levels, especially those aged < 60 years, non-Hispanic white/black individuals, and individuals with a BMI ≥ 25. These findings were consistent with those of previous research (28–30).

Dysregulation of liver vitamin A homeostasis may be implicated in the onset of NAFLD (9). Disordered vitamin A homeostasis during the development and early stages of NAFLD can affect the accumulation of lipids in the liver (31, 32). This results in excessive release of retinol and elevated serum retinol levels (33). When NAFLD develops into advanced fibrosis, excessive impairment of vitamin A levels leads to a serious deficiency of vitamin A in the liver, which leads to decreased serum retinol concentrations.

This study has several limitations. First, the causal relationship between both NAFLD and liver fibrosis and serum retinol levels remains unclear; this should be confirmed by prospective studies. Second, NAFLD status and liver fibrosis were defined by the CAP and LSM values through VCTE rather than liver biopsy, which may cause identification bias. Third, the correlation between both NAFLD and liver fibrosis and serum retinol levels varied by age, sex, race, and BMI in the subgroup analysis; the mechanism remains unclear and needs further research.

## 5. Conclusion

Our study suggests that NAFLD status may be positively associated with serum retinol levels in adult patients and that liver fibrosis may be negatively associated with serum retinol levels. As many unknown and complicated mechanisms exist, future experimental and prospective cohort studies will be helpful.

## Data availability statement

Publicly available datasets were analyzed in this study. This data can be found at: www.cdc.gov/nchs/nhanes/.

## Ethics statement

NHANES was approved by the National Center for Health Statistics Research Ethics Review Board. The participants provided their written informed consent to participate in NHANES. Ethical approval for this study is deemed exempt because this study uses publically available data.

## Author contributions

XN, JL, and KL contributed to the study’s conception and design. XN and JL contributed to data collection and interpretation of data. KL contributed to data analysis and writing and revision of the manuscript. All authors contributed to the article and approved the submitted version.

## Conflict of interest

The authors declare that the research was conducted in the absence of any commercial or financial relationships that could be construed as a potential conflict of interest.

## Publisher’s note

All claims expressed in this article are solely those of the authors and do not necessarily represent those of their affiliated organizations, or those of the publisher, the editors and the reviewers. Any product that may be evaluated in this article, or claim that may be made by its manufacturer, is not guaranteed or endorsed by the publisher.

## Footnotes

^1^ wwwn.cdc.gov

^2^ http://www.empowerstats.com

^3^ http://www.R-project.org
